# Sub-Inhibitory Concentrations of Mupirocin Strongly Inhibit Alpha-Toxin Production in High-Level Mupirocin-Resistant MRSA by Down-Regulating *agr, saeRS*, and *sarA*

**DOI:** 10.3389/fmicb.2018.00993

**Published:** 2018-05-15

**Authors:** Ye Jin, Meilan Li, Yongpeng Shang, Li Liu, Xiaofei Shen, Zhihui Lv, Zhihao Hao, Jingjing Duan, Yang Wu, Chun Chen, Jingye Pan, Fangyou Yu

**Affiliations:** ^1^Department of Laboratory Medicine, The First Affiliated Hospital of Wenzhou Medical University, Wenzhou, China; ^2^Emergency Intensive Care Unit, Shanghai Pulmonary Hospital, Tongji University School of Medicine, Shanghai, China; ^3^Key Laboratory of Medicine Molecular Virology, Ministry of Education and Ministry of Public Health, Shanghai Medical College, Fudan University, Shanghai, China; ^4^Department of Respiratory Medicine, Zhejiang Provincial People’s Hospital, Hangzhou Medical College, Hangzhou, China; ^5^Department of Laboratory Medicine, Shanghai Pulmonary Hospital, Tongji University School of Medicine, Shanghai, China

**Keywords:** MRSA, sub-inhibitory concentrations, mupirocin, alpha-toxin, *agr*, *saeRS*, *sarA*

## Abstract

Mupirocin, a topical antibiotic, has been utilized for decades to treat *Staphylococcus aureus* skin infections, as well as to decolonize patients at risk of methicillin-resistant *S. aureus* (MRSA) infection. The aims of this study were to investigate the expression of α-toxin (encoded by the *hla* gene) in ten clinical MRSA strains (MIC = 1024 μg/ml) in response to a sub-inhibitory concentration of mupirocin (1/32 minimum inhibitory concentration [MIC]) (32 μg/ml) by using α-toxin activity determination and enzyme-linked immune sorbent assay (ELISA). Subsequently, real-time polymerase chain reaction (RT-PCR) was used to examine the expression of *saeR*, *agrA*, *RNAIII*, and *sarA* genes under sub-inhibitory concentration of mupirocin in order to investigate the mechanism of action of this treatment regarding its strong inhibition of α-toxin expression. For all the strains tested, mupirocin dramatically reduced mRNA levels of α-toxin. The results indicated that α-toxin activity in mupirocin-treated groups was significantly lower than that in untreated groups. The results show that the levels of *agrA*, *RNAIII*, *saeR*, and *sarA* expression significantly decrease by 11.82- to 2.23-fold (*P* < 0.01). Moreover, we speculate that mupirocin-induced inhibition of α-toxin expression may be related to the inhibition of regulatory loci, such as *agr*, *sarA* and *saeRS*. More specifically, we found that the mechanism involves inhibiting the expression of *agrA* and *RNAIII*. In conclusion, sub-inhibitory concentrations of mupirocin strongly inhibit alpha-toxin production in high-level mupirocin-resistant MRSA by down-regulating *agr, saeRS* and *sarA*, which could potentially be developed as a supplemental treatment to control high-level mupirocin-resistant MRSA infection and reduce the risk of infection and colonization.

## Introduction

Methicillin-resistant *Staphylococcus aureus* (MRSA) is one of the major causes of hospital- and community-acquired infections, including those associated with burn injury, sepsis, pneumonia, keratitis, and nasosinusitis (David and Daum, 2010). Moreover, colonization with MRSA greatly increases the risk of MRSA infection (Coello et al., 1997; Davis et al., 2004).

**FIGURE 1:**
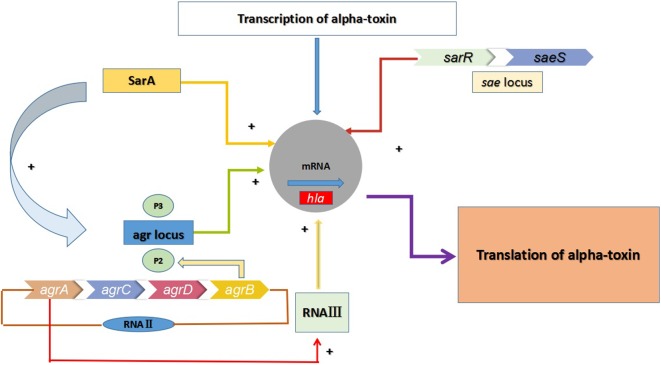
Multiple regulatory factors regulate the expression of *hla*. The *agr* locus encodes two transcripts known as *RNAII* and *RNAIII*, which are transcribed from the P2 and P3 promoters. The regulatory RNA molecule *RNAIII* can up-regulate the expression of *hla*, and *agrA* is an essential transcription factor for *RNAIII*. *RNAII* encodes AgrB, AgrD, AgrC, and AgrA. Meanwhile, the expression of *hla* in MRSA is also regulated by the two-component regulatory system SaeRS (*saeR* and *saeS*)and the SarA protein family.

In the aforementioned infections, alpha-toxin (α-toxin) plays an indispensable role in the pathogenicity of *S. aureus* by causing tissue damage. α-toxin is a 33.2-kDa extracellularly secreted protein encoded by the *hla* gene (Bhakdi and Tranum-Jensen, 1991). It is a pore-forming toxin that causes damage and death of cells (Bhakdi and Tranum-Jensen, 1991). This protein has been documented as a virulence factor in many severe infections, including keratitis, mastitis, nasosinusitis, peritonitis, and skin and soft tissue infections (SSITs) (Bayer et al., 1997). As shown in **Figure 1**, three regulatory systems, comprising the accessory gene regulator (*agr*), staphylococcal accessory protein effector (*saeRS*) and staphylococcal accessory gene regulator (*sarA*) systems, appear to regulate *hla* expression in a coordinated manner *in vitro* (Xiong et al., 2006). The *agr* locus activates *hla* expression directly and positively, while *sarA* exerts a positive impact on *hla* expression by both *agr*-dependent and *agr*-independent pathways. In addition, the sae locus includes a two-component signal-transduction system encoded by *saeS* and *saeR* that positively regulates the expression of *hla* at the transcriptional level. Moreover, *sae* activation is also affected by *agr*, as well as by some stress environment.

Mupirocin (pseudomonic acid A), a competitive inhibitor of isoleucyl-tRNA synthetase (IRS), is an active antibiotic against most of gram-positive bacteria (Fuller et al., 1971; Thomas et al., 2010). It mediates the inhibition of ribosomal IRS binding, thereby impeding protein and RNA synthesis and leading to cell death (Hughes and Mellows, 1978). Mupirocin first became available in 1985, and it has been widely used for the management of topical MRSA infection and colonization in both patients and healthcare personnel (Boelaert, 1994).

Two categories of mupirocin resistance are recognized: high-level, plasmid-mediated resistance (minimum inhibitory concentration [MIC] ≥512 μg/ml) and low-level, chromosomally encoded resistance (MIC ≤256 μg/ml) (Eltringham, 1997). In recent years, various studies have shown that high-level mupirocin resistance is associated with MRSA colonization and treatment failure (Simor, 2011; Poovelikunnel et al., 2015), partially due to the inability to attain mupirocin concentrations ≥512 μg/ml in clinical settings. However, as previous reports have shown, sub-inhibitory concentrations of antibacterial agents can modulate the expression of virulence factors in *S. aureus* (Ohlsen et al., 1998; Herbert et al., 2001; Deneve et al., 2009; Otto et al., 2013; Li et al., 2014), and thereby impact the outcome of severe infections. Despite this, limited work has been undertaken to investigate the effect of sub-inhibitory concentrations of mupirocin on virulence factors produced by *S. aureus*.

In this study, therefore, we investigated the hypothesis that sub-inhibitory concentrations of mupirocin can prevent the release of the virulence factors, such as α-toxin.

## Materials and Methods

### Bacterial Strains

All ten clinical mupirocin-resistant *S. aureus* strains (SA001–SA010) in this study were isolated from the First Affiliated Hospital of Wenzhou Medical University located in Wenzhou, east China, from 2013 to 2016 (**Table 1**). Identification and antimicrobial susceptibility testing of the isolates were carried out using a Vitek-2 Microbiology Analyzer (bioMerieux, Marcy l’Etoile, France) in accordance with the manufacturer’s instructions. The *S. aureus* ATCC25923 was used as the control strain for the identification of bacterial clinical isolates.

**Table 1:** Strains used in this study.

Strains	ST	SCC*mec*	*mecA*	MIC (μg/ml)	*mupA*	Ward	Year	Source
SA001	7	Unknown	+	1024	+	Neurology	2013	Sputum
SA002	5	II	+	1024	+	ICU	2014	Blood
SA003	5	II	+	1024	+	Operation room	2015	Skin
SA004	239	III	+	1024	+	Temporary turnover ward	2016	Sputum
SA005	1	IV	+	1024	+		2015	Pus
SA006	59	IVa	+	1024	+	Neurology ward	2013	Sputum
SA007	121	II	+	1024	+	ICU	2014	Blood
SA008	7	II	+	1024	+	ICU	2015	Sputum
SA009	5	II	+	1024	+	Urinary surgery ward	2016	Skin
SA010	764	Unknown	+	1024	+	Brain intensive care ward		Pus

*Sequence types (ST) and Sccmec types distribution the source among S. aureus clinical isolates.*

### MRSA Identification

Polymerase chain reaction was used to detect whether the strains tested harbored *mecA* and *mecC*, with MRSA N315 as the positive control strain. The strains carrying *mecA* or *mecC* were defined as MRSA. Moreover, all the clinical strains with high-level mupirocin resistance were targeted *mupA* by PCR assays.

### Multilocus Sequence Typing (MLST)

Multilocus sequence typing of the *S. aureus* strains was determined by amplifying seven housekeeping genes (*arc*, *aroE*, *glpF*, *gmk*, *pta*, *tpi*, and *yqiL*) as described previously (Saunders and Holmes, 2007). The sequences were compared with the existing sequences available on the online database^1^, and sequence types (STs) were assigned according to the allelic profiles.

### Staphylococcal Cassette Chromosome *mec* (SCC*mec*) Typing

Staphylococcal cassette chromosome *mec* typing of all the high-level mupirocin-resistant strains was determined by multiplex PCR with eight unique and specific primer pairs for SCC*mec* I, II, III, IVa, IVb, IVc, IVd, and V, as described previously (Zhang et al., 2005).

### Mupirocin MIC Determination

Mupirocin was obtained from Sigma-Aldrich (St. Louis, MO, United States). MICs were determined with the standard broth microdilution method recommended by the Clinical and Laboratory Standards Institute [CLSI] (2017). The *S. aureus* ATCC 29213 was used as the control strain in accordance with CLSI breakpoints (Clinical and Laboratory Standards Institute [CLSI], 2017). Thereafter, tryptic soy broth (TSB; Sigma-Aldrich) culture medium containing either 1/8 MIC (128 μg/ml), 1/16 MIC (64 μg/ml), 1/32 MIC (32 μg/ml), or 1/64 MIC (16 μg/ml) of mupirocin, or no mupirocin, was prepared for each strain to measure virulence factor expression.

### α-Toxin Activity Determination

The strains tested were grown in TSB (Sigma-Aldrich) at 37°C overnight. After 24 h of incubation, the bacteria were diluted to a concentration 5 × 10^5^ CFU/ml. Different concentrations of mupirocin were then each added to the TSB. Overnight cultures containing TSB with 1/8, 1/16, 1/32, and 1/64 MIC of mupirocin or without mupirocin were normalized to the same optical density values at 600 nm (OD_600_) and then centrifuged at 4000 × *g* to harvest the bacterial cells. Aliquots of the supernatants were added to a 1% suspension of washed rabbit red blood cells (RRBCs) in 0.01 M phosphate-buffered saline (PBS; pH 7.2) containing 0.1% bovine serum albumin (Sigma-Aldrich), and they were then incubated for 1 h at 37°C. We used Triton X-100 as a positive control and RRBCs with 0.9% NaCl solution as a negative control. The absorbance at 600 nm (A_600_) of the complete hemolysis group (positive control) is set to 100. The A_600_ percentage experimental group is the ratio of A_600_ to complete hemolysis groups in each group and multiplied by 100. All data have been calibrated with negative controls. Each test was performed independently in triplicate.

### Quantitative Enzyme-Linked Immunosorbent Assay (ELISA) for α-Toxin

The strains tested were cultured at 37°C in 96-well microtiter plates containing TSB with 1/32 MIC of mupirocin or without mupirocin overnight. Aliquots of each culture were then centrifuged at 4,000 × *g* for 10 min. The α-toxin level in the supernatant was then assessed. The concentration of α-toxin in the supernatant was quantified using a specific sandwich-type ELISA (Sigma-Aldrich) targeting *hla* with an anti-*hla* monoclonal antibody as primary antibodies. We then detected the antibody–antigen complex by using an anti-*hla* polyclonal rabbit antibody and a peroxidase-conjugated goat anti-rabbit antibody as secondary antibodies. The concentration of α-toxin in each sample was calculated using the linear regression method with a standard curve, *y* = *ax*+*b*. The absorbance readings were obtained by subtracting the absorbance of the blank from the absorbance of the samples subjected to ELISA. Untreated supernatant was used as the negative control. The cut-off value was defined as less than twice the value of the negative control absorbance.

### Real-Time Polymerase Chain Reaction (RT-PCR)

A total of 4 ml of bacterial culture (grown with 1/32 MIC of mupirocin, as well as without mupirocin) was incubated at 37°C with shaking at 220 rpm for 16 h. Each solution was centrifuged at 12000 × *g* for 2 min. After centrifugation, the supernatant was discarded. We then added each pellet plus 1 ml 0.1-mm zirconia-silica beads to a vial tube and filled it with normal saline without bubbles. The tube was then shaken at 4000 rpm for 1 min using a Mini-Bead Beater (BioSpec Products, Bartlesville, OK, United States), followed by 1 min of cooling on ice. This procedure was repeated for five times.

The total RNA was then purified using an RNeasy Plus Mini Kit (QIAGEN, Berlin, Germany) following the manufacturer’s instructions. The purified RNA (1 μg) was used to generate cDNA using a PrimeScript RT Reagent Kit (TaKaRa, Tokyo, Japan), and the resulting cDNA was amplified using a SYBR Green Premix Kit (TaKaRa, Tokyo, Japan) with a Mastercycler ep realplex instrument (Eppendorf, Hamburger, Germany). The *hla* gene and regulatory genes (*agr*, *sarA*, *saeR*, and *RNAIII*) were determined by RT-PCR. **Table 2** shows the oligonucleotide primers. Cultures of the *S. aureus* strains grown without mupirocin were used as positive controls (relative expression = 1), and *gyrB* was used as an endogenous control to investigate target genes. RNA transcript levels were calculated using the expression 2^-ΔΔ*C*_T_^. Each reaction was performed in triplicate.

**Table 2:** Primers used in this study.

Primer	Sequence (5′ → 3′)
*Gyrb-F*	*GGTGGCGACTTTGATCTAGC*
*Gyrb-R*	*TTATACAACGGTGGCTGTGC*
*RT-hla-F*	*GGTATATGGCAATCAAC*
*RT-hla-R*	*CTCGTTCGTATATTACATCT*
*RT-agrA-F*	*TCCAGCAGAATTAAGAACTCG*
*RT-agrA-R*	*ATATCATCATATTGAACATACACT*
*RT-saeR-F*	*CGTCCTCGTCACTTTGTTGA*
*RT-saeR-R*	*ATCGTGGATGATGAACA*
*RT-sarA-F*	*AAACCCTGAATTTGAATG*
*RT-sarA-R*	*GATATTACATCTGCTCCT*
*RT-RNAIII-F*	*GCACTGAGTCCAAGGAAACTAAC*
*RT-RNAIII-R*	*AAGCCATCCCAACTTAATAACC*

### Statistical Analysis

Chi-square or Fisher’s exact tests were used to compare the differences in α-toxin activity and *hla* expression among the groups treated with 1/8 to 1/32 MIC of mupirocin or no mupirocin. SPSS statistical software (version 19, IBM Corp, Armonk, NY, United States) was used, and a 2-sided *p*-value <0.05 was considered statistically significant. The ELISA and RT-PCR data were analyzed using GraphPad Prism software (version 7.00, La Jolla, CA, United States), and a *p*-value <0.05 was considered statistically significant.

## Results

### Strains Information

The details of the strains used in this study are described in **Table 1**. All clinical isolates were isolated from different wards, comprising the intensive care unit (ICU), nephrology ward, temporary turnover ward, brain intensive care ward, urinary surgery ward, and operation room. The sources of the isolates comprised sputum, blood, pus, and skin. All clinical isolates possessed high-level resistance to mupirocin (MIC = 1024 μg/ml) and were positive for *mupA*. Regarding the molecular characteristics, the SA001–SA004 were identified as ST5-MRSA-II, ST239-MRSA-III, ST7-MRSA-un, and ST5-MRSA-II, respectively. The information of strains SA005–SA010 was shown in **Table 1**.

### Effect of Sub-Inhibitory Concentrations of Mupirocin on α-Toxin Activity

As it is difficult to achieve the concentrations of mupirocin >256 μg/ml (1/4MIC7) in serum, we explored the effects of mupirocin on α-toxin expression using concentrations of 16–128 μg/ml (1/64 to 1/8 MIC).

**FIGURE 2:**
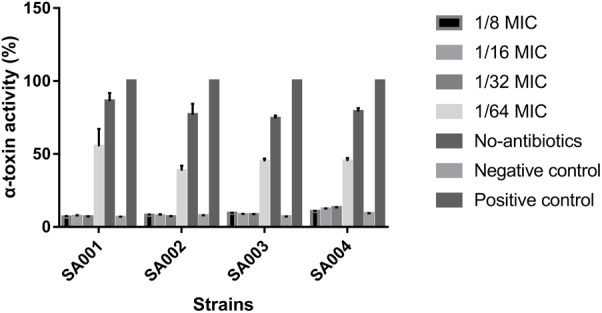
Effects of sub-inhibitory concentrations of mupirocin (1/64–1/8 MIC) on α-toxin activity in high-level mupirocin-resistant MRSA strains. We used Triton X-100 (which causes complete hemolysis) as a positive control and RRBCs with 0.9% NaCl solution as a negative control. The absorbance at 600 nm (A600 nm) of the positive control was set to 100. The α-toxin activity percentage for each experimental group is the ratio of the A600 nm for that group to the A600 nm of the positive control multiplied by 100. All data were calibrated with negative controls. Each test was performed independently in triplicate. Values are means ± SD (three repeated experiments).

With respect to observed differences in α-toxin activity between untreated strains and strains exposed to 1/64 MIC (16 μg/ml), 1/32 MIC (32 μg/ml), 1/16 MIC (64 μg/ml), and 1/8 MIC (128 μg/ml) mupirocin, we found that α-toxin activity among the latter groups was significantly reduced (*P* < 0.05) except for the 1/64 MIC group. When cultured without mupirocin, 74.26 – 88.6% (SA001–SA004) of the RRBCs were lysed by α-toxin (**Figure 2**). When cultured in the presence of 1/8 MIC mupirocin, these percentages significantly decreased (*P* < 0.05) to 7.20 – 10.74%, respectively. When cultured with 1/16 and 1/32 MIC mupirocin, they also decreased significantly compared with the untreated strains (*P* < 0.05) (data not shown). However, 1/64 MIC group did not produce a significant decrease in α-toxin activity (*P* > 0.05). In summary, compared with culture without mupirocin, mupirocin with 1/8, 1/16 and 1/32 MIC induced significant reductions in α-toxin activity, resulting in 13.03- to 6.44-fold in strains SA001–SA004. The results show that mupirocin treatment is concentration-independent. As a result, regarding strains SA005–SA010, we investigated the effects of mupirocin on α-toxin expression using concentration of 32 μg/ml (1/32 MIC). As shown in **Supplementary Figure S1**, when cultured with 1/32 MIC, the treatment group shows significant decrease (*P* < 0.05) compared with the untreated group.

### Effect of Sub-Inhibitory Concentrations of Mupirocin on α-Toxin Production

**FIGURE 3:**
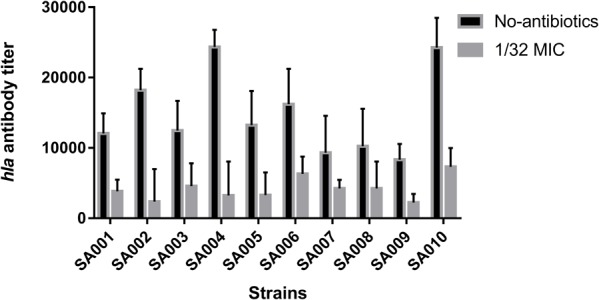
Effects of a sub-inhibitory concentration of mupirocin on α-toxin release. All the high-level mupirocin-resistant clinical isolates (SA001–SA010) were cultured with mupirocin at 1/32 MIC or without mupirocin. The α-toxin concentration in the supernatant was quantified by ELISA. The α-toxin concentration in each sample was calculated using the linear regression method with a standard curve, *y = ax+b*. The absorbance readings were obtained by subtracting the absorbance of the blank from the absorbance of the samples subjected to ELISA. Untreated supernatant was used as the negative control. The cut-off value was defined as less than twice the value of the negative control absorbance.

As described above, we conclude that mupirocin can reduce α-toxin production in the clinical strains SA001–SA010. To investigate this further, we used ELISA to investigate the nature of α-toxin production in *S. aureus* when cultured with or without sub-inhibitory concentrations of mupirocin (1/32 MIC). As shown in **Figure 3**, exposure to sub-inhibitory concentrations of mupirocin resulted in large decreases in α-toxin production, with antibody titers 7.74–2.20-fold lower than those of cultures not exposed to mupirocin. However, the 1/64 MIC of mupirocin is not as strongly not responding to α-toxin secretion (*P* > 0.05). These findings demonstrate that 1/32 MIC of mupirocin can greatly reduce external α-toxin secretion. As a result, to analyze the degree of production further, we finally selected a certain concentration of mupirocin (1/32 MIC) to explore the effects of this sub-inhibitory concentration of mupirocin on α-toxin.

### Effect of a Sub-Inhibitory Concentration of Mupirocin on *hla* Expression

**FIGURE 4:**
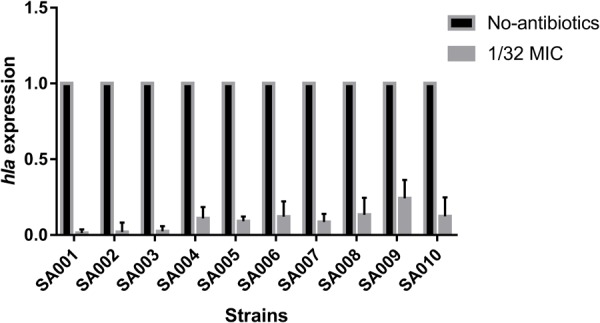
Real-time polymerase chain reaction (RT-PCR) analysis of *hla* expression after exposure to a sub-inhibitory concentration of mupirocin (1/32 MIC). All the high-level mupirocin-resistant clinical isolates (SA001–SA010) were cultured with mupirocin at 1/32 MIC or without mupirocin. The results are expressed as n-fold differences in the *hla*/*gyrB* ratio in the presence of mupirocin relative to the *hla/gyrB* ratio without mupirocin. Values are means+SDs (based on three repeated assays). There were significant differences with the control group (grown without mupirocin) for each strain (*P* < 0.05).

To explore the influence of a sub-inhibitory concentration (1/32 MIC) of mupirocin on *hla* expression, we performed relative quantitative PCR to assess the *hla*/*gyrB* ratios in cultures grown with and without mupirocin for 16 h. As expected, mupirocin induced a strong decrease of *hla* mRNA levels in strains SA001–SA010 after 16 h incubation. As shown in **Figure 4**, when cultured with 1/32 MIC of mupirocin compared with culture without mupirocin, the level of *hla* expression significantly (*P* < 0.05) decreased by 73.52–4.12-fold depending on the strain (see the concrete data in **Supplementary Table S1**). Therefore, when considering these concentrations together (1/8, 1/16 and 1/32 MIC), we can conclude that mupirocin significantly decrease the α-toxin level in the bacterial external secretion by affecting the expression of the *hla* gene.

### Effects of a Sub-Inhibitory Concentration of Mupirocin on the Expression of Regulatory Genes *agr* (*agrA and RNAIII*), *saeRS* (*saeR*), and *sarA*

**FIGURE 5:**
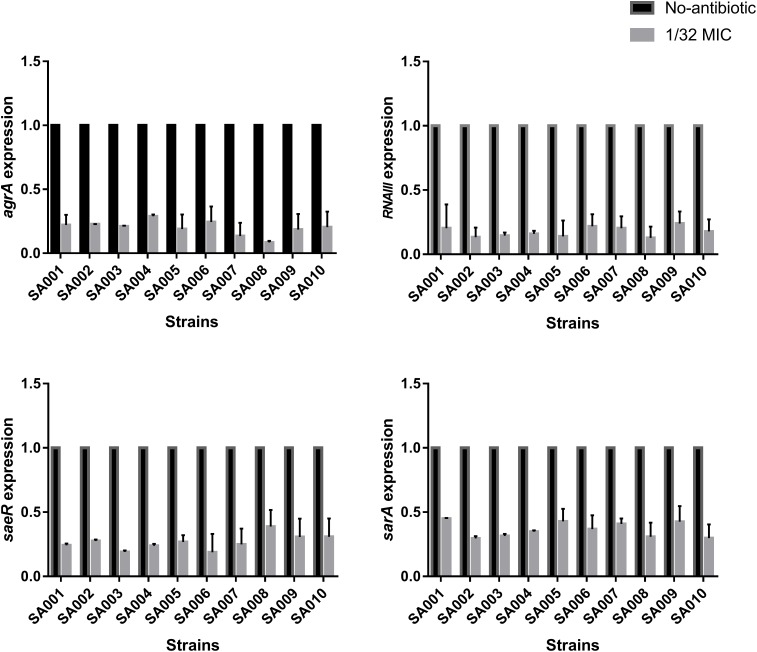
Effects of sub-inhibitory a concentration of mupirocin (1/32 MIC) on the expression of regulatory genes. All the high-level mupirocin-resistant clinical isolates (SA001–SA010) were cultured with mupirocin at 1/32 MIC or without mupirocin. The results are expressed as n-fold differences in the *RNAIII*/*gyrB*, *sarA*/*gyrB*, *agrA*/*gyrB*, and *saeR*/*gyrB* ratios in the presence of mupirocin relative to the same ratios of the strain grown without mupirocin. Values are means+SDs (based on three repeated assays). There were significant differences with the control group (grown without mupirocin) for each strain (*P* < 0.05).

We have shown that sub-inhibitory concentrations of mupirocin reduce the level of α-toxin in *S. aureus*. To elucidate the inhibition mechanism of mupirocin on *hla* mRNA expression, we investigated the expression of the major virulence regulatory genes in *S. aureus* (*agr*, *saeRS*, and *sarA*) under the same conditions. As shown in **Figure 5**, when the strains were clutured with 1/32 MIC of mupirocin for 16 h, the levels of *agrA* expression significantly decrease (*P* < 0.01) by 11.82- to 3.46-fold (*P* < 0.01). The levels of *RNAIII* expression significantly decrease (*P* < 0.01) by 7.80- to 3.46-fold (*P* < 0.01). About the levels of *saeR* and *sarA*, the expressions significantly decrease by 2.58- to 5.32-fold (*P* < 0.01) and 3.38- to 2.23-fold (*P* < 0.01) respectively (see the concrete data in **Supplementary Table S1**). These results demonstrate that sub-inhibitory concentrations of mupirocin most likely act by repressing the expression of these regulatory genes, especially at the *agr* regulatory locus.

## Discussion

The rapid global dissemination of antibiotic-resistant *S. aureus* strains has rendered treatment of serious infections increasingly difficult (Tong et al., 2015). As a result, a number of studies have focused on the effects of sub-inhibitory concentrations of antibiotics on bacterial cell functions, including virulence factor expression (Otto et al., 2013). In this study, we first investigated the effect of sub-inhibitory concentrations of mupirocin on *hla* expression, utilizing clinical MRSA isolates that exhibit high-level mupirocin resistance.

The clinical performance of antibiotics is usually evaluated based firstly on their bactericidal or bacteriostatic effect and secondly on their impact on the release of virulence factors. Moreover, sub-inhibitory concentrations of antibiotics against *S. aureus* have been shown to interfere with the expression of virulence factors such as regulatory gene products that in turn affect the transcription of exoprotein-encoding genes (Otto et al., 2013). Recently, the effects of sub-inhibitory concentrations of β-lactams and fluoroquinolones on the expression of virulence factors in pathogenic Gram-positive bacteria have been investigated by several studies (Otto et al., 2013). These studies have demonstrated that the changes of virulence factor expression induced by different sub-inhibitory concentrations of antibiotics are diverse, and can be detected by different methods (Davies et al., 2006). In *S. aureus*, exposure to different sub-inhibitory concentrations of antibiotics (such as β-lactams, macrolides, aminoglycosides, glycopeptides, and fluoroquinolones) leads to varying degrees of *hla* expression (Ohlsen et al., 1998). More precisely, sub-inhibitory concentrations of β-lactams result in almost complete inhibition of α-toxin production (Otto et al., 2013), while macrolides and aminoglycosides result in partial inhibition (Ohlsen et al., 1998), glycopeptides have no effect, and fluoroquinolones lead to slightly increased expression (Ohlsen et al., 1998; Otto et al., 2013).

In our study, to detect α-toxin expression, we first calculated mupirocin MIC values using broth microdilution according to CLSI breakpoints. After confirming the mupirocin MICs, we chose 1/32 MIC (32 μg/ml) as a suitable concentration of mupirocin for culturing *S. aureus* strains for the remaining experiments. Based on the results, we conclude that sub-inhibitory concentrations of mupirocin lead to reductions of α-toxin production by suppressing the expression of *hla*. To confirm the phenotypic effect, we first investigated the α-toxin activity for each group, demonstrating that *hla* activity of the mupirocin-untreated groups was significantly lower than that of the untreated group. ELISA was then used to confirm the degree of α-toxin production. To explore what caused the decrease of *hla* expression, we determined the effects of a sub-inhibitory concentration of mupirocin on *hla* mRNA levels.

As a topical antimicrobial agent, mupirocin mediates the inhibition of isoleucyl-tRNA synthetase (IRS), thereby impeding protein and RNA synthesis (Fuller et al., 1971). The expression of virulence factors is controlled in a coordinated fashion by a network of regulatory systems, such as *agr*, *sarA*, and *saeRS*. The most likely mechanism for decreased production of exocrine proteins (such as α-toxin) involves blocking mRNA translation at the ribosomal level. Moreover, it is tempting to speculate that mupirocin-induced inhibition of *hla* may result from the suppression of regulatory systems such as *agr*, *saeRS*, and *sarA*. The *agr* locus encodes two transcripts known as *RNAII* and *RNAIII*, which are transcribed from the P2 and P3 promoters (Morfeldt et al., 1996). The regulatory RNA molecule *RNAIII* can up-regulate the expression of virulence genes such as *hla*, and *agrA* is an essential transcription factor for *RNAIII*. Meanwhile, the expression of *hla* in MRSA is also regulated by the two-component regulatory system SaeRS and the SarA protein family. Therefore, we speculate that sub-inhibitory concentrations of mupirocin strongly inhibit *hla* expression in high-level mupirocin-resistant MRSA by down-regulating a network of regulatory systems, namely, *agr*, *saeRS*, and *sarA*.

As a result, we demonstrate that sub-inhibitory concentrations of mupirocin can reduce the expression of the virulence gene *hla* in high-level mupirocin-resistant clinical strains. We find that sub-inhibitory concentrations of mupirocin reduce *hla* expression by interfering with at least three key regulatory loci: *agr*, *saeRS*, and *sarA*. We successfully show that reduction in *RNAIII* and *agrA* expression, and RNAIII-controlled virulence factors, is a result of a direct or indirect interaction between mupirocin and *hla*. Thus, mupirocin may modulate *hla* expression by blocking Agr’s ability to bind to the P2-P3 promoter region of the *agr* locus. Furthermore, we find that mupirocin plays a role in the inhibition of *saeRS* and *sarA*, which is essential to the pathogenicity of *S. aureus*. Although the exact mechanisms involved with the inhibition of these key virulence traits by mupirocin remain to be unascertained, we demonstrate that a sub-inhibitory concentration of mupirocin can be an efficient repressor of virulence gene expression.

This conjecture was supported by the findings that, when treated with a sub-inhibitory concentration of mupirocin, the expression levels of *RNAIII, agrA*, *saeR*, and *sarA* were all significantly reduced. Several investigators have reported that sub-inhibitory concentrations of clindamycin result in almost complete inhibition of α-toxin (Ohlsen et al., 1998; Herbert et al., 2001; Otto et al., 2013), while others have reported that *agr* can not be responsible for the clindamycin effect, although *saeRS* could conceivably have a role with respect to certain exoproteins (Herbert et al., 2001). In contrast, in our study, *agrA*, *RNAIII, saeR*, and *sarA* expression levels were all significantly reduced following exposure to a sub-inhibitory concentration of mupirocin. However, regarding the inhibition mechanism of action of sub-inhibitory concentrations of mupirocin, there are a lot work to be done.

## Conclusion

In conclusion, in this study, we demonstrate mupirocin can suppress alpha-toxin in a dose-independent manner by phenotypic and transcriptional expression analysis. In addition, the findings in our study indicate that sub-inhibitory concentrations of mupirocin may be of great importance as supplemental treatment for controlling high-level mupirocin-resistant MRSA infection and preventing infection and colonization, as this treatment could be used as a targeted-therapy against α-toxin.

## Author Contributions

YJ, ML, YS, ZH, LL, JD, and XS designed of the work and analyzed and interpreted of data for the work. FY and CC drafted the work and revised it critically for important intellectual content. JP provided approval for publication of the content. YS, ZL, and YW participated in the experimental design and data analysis. FY agreed to be accountable for all aspects of the work in ensuring that questions related to the accuracy or integrity of any part of the work are appropriately investigated and resolved. All authors read and approved the final manuscript.

## Conflict of Interest Statement

The authors declare that the research was conducted in the absence of any commercial or financial relationships that could be construed as a potential conflict of interest.

## Footnotes

1 www.mlst.net

## Supplementary Material

FIGURE S1 Effects of a sub-inhibitory concentration of mupirocin (1/32 MIC) on α-toxin activity in high-level mupirocin-resistant MRSA strains. We used Triton X-100 (which causes complete hemolysis) as a positive control and RRBCs with 0.9% NaCl solution as a negative control. The absorbance at 600 nm (A600nm) of the positive control was set to 100. The α-toxin activity percentage for each experimental group is the ratio of the A600nm for that group to the A600nm of the positive control multiplied by 100. All data were calibrated with negative controls. Each test was performed independently in triplicate. Values are means ± ±SD (three repeated experiments). ^∗^Significantly different from the control (strain grown without mupirocin).

TABLE S1 The reduction coefficient (1/32 MIC) of α-toxin activity and the transcriptional levels of *hla, RNAIII, agrA, saeR*, and *sarA* (compared to the group without mupirocin).
