# Supplementary figures and images for: Sub-Inhibitory Concentrations of Mupirocin Strongly Inhibit Alpha-Toxin Production in High-Level Mupirocin-Resistant MRSA by Down-Regulating agr, saeRS, and sarA
Source: Front Microbiol. 2018 May 15;9:993. doi: 10.3389/fmicb.2018.00993 (PMC5962727; doi:10.3389/fmicb.2018.00993)

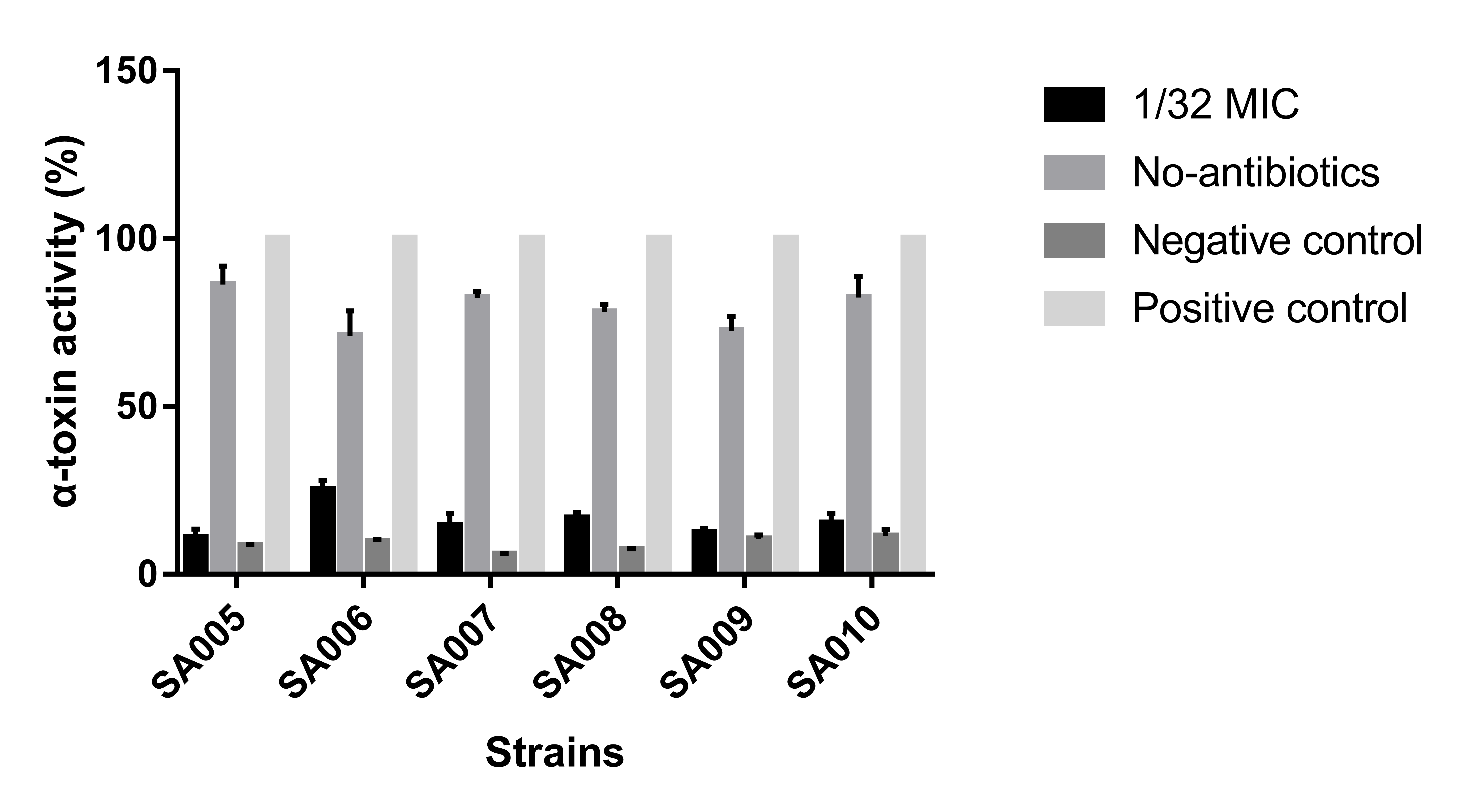

Supplement: FIGURE S1 — Effects of a sub-inhibitory concentration of mupirocin (1/32 MIC) on α-toxin activity in high-level mupirocin-resistant MRSA strains. We used Triton X-100 (which causes complete hemolysis) as a positive control and RRBCs with 0.9% NaCl solution as a negative control. The absorbance at 600 nm (A600nm) of the positive control was set to 100. The α-toxin activity percentage for each experimental group is the ratio of the A600nm for that group to the A600nm of the positive control multiplied by 100. All data were calibrated with negative controls. Each test was performed independently in triplicate. Values are means ± ±SD (three repeated experiments). ∗Significantly different from the control (strain grown without mupirocin). [file Image_1.tif]
