# Supplementary material for: Sub-Inhibitory Concentrations of Mupirocin Strongly Inhibit Alpha-Toxin Production in High-Level Mupirocin-Resistant MRSA by Down-Regulating agr, saeRS, and sarA
Source: Front Microbiol. 2018 May 15;9:993. doi: 10.3389/fmicb.2018.00993 (PMC5962727; doi:10.3389/fmicb.2018.00993)
Supplement: TABLE S1 — The reduction coefficient (1/32 MIC) of α-toxin activity and the transcriptional levels of hla, RNAIII, agrA, saeR, and sarA (compared to the group without mupirocin). [file Data_Sheet_1.docx]

**Supplementary materials Table 1.** The reduction coefficient (1/32 MIC) of α-toxin activity, the transcriptional level of *hla, RNAIII, agrA, saeR* and *sarA* (compared to no-mupirocin group).

| **Strains** | **Reduction coefficient** | | | | | | |
| --- | --- | --- | --- | --- | --- | --- | --- |
|  | α-toxin activity | *hla* antibody titer | *hla* level | *agrA* level | *RNAIII* level | *saeR* level | *sarA* level |
| SA001 | 12.46 | 3.14 | 73.52 | 4.53 | 4.91 | 4.14 | 2.23 |
| SA002 | 11.07 | 7.74 | 51.01 | 4.41 | 7.53 | 3.62 | 3.38 |
| SA003 | 8.74 | 2.73 | 41.71 | 4.74 | 6.85 | 5.23 | 3.18 |
| SA004 | 5.94 | 7.53 | 9.17 | 3.46 | 6.28 | 4.20 | 2.87 |
| SA005 | 7.98 | 4.03 | 11.08 | 5.29 | 7.18 | 3.73 | 2.33 |
| SA006 | 2.83 | 2.57 | 8.31 | 4.09 | 4.58 | 5.32 | 2.71 |
| SA007 | 5.71 | 2.20 | 11.74 | 7.43 | 4.93 | 4.02 | 2.45 |
| SA008 | 4.66 | 2.41 | 7.53 | 11.82 | 7.80 | 2.58 | 3.24 |
| SA009 | 5.85 | 3.71 | 4.12 | 5.38 | 4.18 | 3.25 | 2.35 |
| SA010 | 5.40 | 3.31 | 8.09 | 4.89 | 5.61 | 3.25 | 3.35 |
